# Prevalence of bacterial genotypes and outcome of bovine clinical mastitis due to *Streptococcus dysgalactiae* and *Streptococcus uberis*

**DOI:** 10.1186/s13028-014-0080-0

**Published:** 2014-11-27

**Authors:** Åsa Lundberg, Ann Nyman, Helle Ericsson Unnerstad, Karin Persson Waller

**Affiliations:** Department of Animal Health and Antimicrobial Strategies, National Veterinary Institute, SE-751 89 Uppsala, Sweden; Department of Clinical Sciences, Swedish University of Agricultural Sciences, SE-750 07 Uppsala, Sweden

**Keywords:** *Streptococcus dysgalactiae* subsp. *dysgalactiae*, *Streptococcus uberis*, Dairy cow, Veterinary-treated clinical mastitis, Genotypes, Somatic cell count, Long-term mastitis outcome, Pulsed-field gel electrophoresis, PFGE

## Abstract

**Background:**

*Streptococcus dysgalactiae* and *Streptococcus uberis* are common causes of clinical mastitis (CM) in dairy cows. In the present study genotype variation of *S. dysgalactiae* and *S. uberis* was investigated, as well as the influence of bacterial species, or genotype within species, on the outcome of veterinary-treated CM (VTCM). Isolates of *S. dysgalactiae* (n = 132) and *S. uberis* (n = 97) were genotyped using pulsed-field gel electrophoresis. Identical banding patterns were called pulsotypes. Outcome measurements used were cow composite SCC, milk yield, additional registered VTCMs and culling rate during a four-month follow-up period.

**Results:**

In total, 71 *S. dysgalactiae* pulsotypes were identified. Nineteen of the pulsotypes were isolated from more than one herd; the remaining pulsotypes were only found once each in the material. All *S. uberis* isolates were of different pulsotypes. During the follow-up period, the SCC of *S. dysgalactiae*-cows was significantly lower than the SCC of *S. uberis*-cows (*P* <0.05). Median SCC of *S. dysgalactiae*-cows was 71 500 cells/ml and of *S. uberis*-cows 108 000 cells/ml. No other differences in outcome parameters could be identified between species or genotypes.

**Conclusions:**

Identical *S. dysgalactiae* genotypes were isolated from more than one herd, suggesting some spread of this pathogen between Swedish dairy herds. The genetic variation among *S. uberis* isolates was substantial, and we found no evidence of spread of this pathogen between herds. The milk SCC was lower during the follow-up period if *S. dysgalactiae* rather than *S. uberis* was isolated from the case, indicating differences in treatment response between bacterial species.

**Electronic supplementary material:**

The online version of this article (doi:10.1186/s13028-014-0080-0) contains supplementary material, which is available to authorized users.

## Background

Clinical mastitis (CM) is a common disease among dairy cows. A number of pathogens can cause CM, but the relative importance of different pathogens differs between regions and countries in the world. In Sweden, *Streptococcus dysgalactiae* subsp. *dysgalactiae* and *Streptococcus uberis* are the third and fourth most common bacteria found in CM accounting for 15.6% and 11.1%, respectively, of the cases [1].

Since the introduction of molecular bacteriology in the field of mastitis, new possibilities for studying the epidemiology of udder pathogens have emerged. *S. uberis* has been extensively studied at the molecular level, and pulsed-field gel electrophoresis (PFGE) has been used for genotypic characterization of *S. uberis* isolates from Europe [2], Australia [3-6], New Zealand [7], and South America [8]. A number of studies have confirmed that this pathogen predominantly shows a heterogeneous genotype pattern consistent with environmentally spread bacteria [2,3,5-7], although there is evidence that *S. uberis* also can be spread between cows as a contagious pathogen within a herd [4,9-11]. There are a few studies reporting that the same genotype can be identified in more than one herd [4,10] indicating possible contagious spread with cows or equipment between herds. Molecular typing has also revealed differences between *S. uberis* strains in clinical manifestation [4,12] and duration of infection [13].

Although often considered as an environmental pathogen [14,15], studies have also indicated that *S. dysgalactiae* may be considered a contagious pathogen [16,17]. Compared with *S. uberis*, only a few studies on genotyping of *S. dysgalactiae* isolates have been performed to our knowledge [5,6,18]. Within a herd, more than one strain is usually present, but a few strains are often found in multiple cows suggesting spread between cows [5,6]. In addition, genetically related isolates have been found on multiple farms, suggesting either contagious spread between herds [6] or a common environmental source [5].

Prudent use of antimicrobials to treat CM is an important part of mastitis control programs. Common aims of treatment are clinical and bacteriological cure, and a return to normal milk somatic cell count (SCC). Bacteriological cure rate have in field studies been reported to be 65–90% for *S. dysgalactiae* and 45–90% for *S. uberis* when using treatment with benzyl penicillin or related compounds [19-21]. Detailed information on clinical cure rate is scarce, but has been reported to be 73% for *S. dysgalactiae* and 77% for *S. uberis* in one of the studies [19]. In a study on treatment of heifers for CM caused by *S. dysgalactiae* around calving, 15.4% of the quarters were nonfunctional and another 36.3% had an increased SCC in the milk and/or an intramammary infection 30 days after treatment [22]. The studies cited above used a follow-up period of two to four weeks after the end of treatment. To our knowledge, studies on differences in treatment outcome between *S. dysgalactiae* and *S. uberis* over a longer time period, or between genotypes within those species, have not been performed.

The understanding of infection epidemiology is essential to prevent and control mastitis. The distribution of *S. dysgalactiae* and *S. uberis* and genotypes within species may vary between regions and countries [23,24], but genotyping studies of those udder pathogens have, to our knowledge, not been performed in the Nordic countries, and not on a national level. The main aim of this study was, therefore, to explore the genotype variation of *S. dysgalactiae* and *S. uberis* associated with bovine CM in Sweden using streptococcal isolates collected in a national survey on CM. The study also aimed to investigate if bacterial species, or genotype within species, influences the outcome of veterinary-treated CM (VTCM), as measured by cow composite SCC, milk production, additional registered VTCMs and culling rate during a four month follow-up period. Differences in cow factors, geography and seasonality between CM cases due to *S. dysgalactiae* and *S. uberis* were also studied.

## Methods

### Bacterial isolates

Isolates of *S. dysgalactiae* (n = 164) and *S. uberis* (n = 117) were collected in a national survey on the prevalence of udder pathogens in bovine CM [1]. Case selection and diagnostic procedures have been described elsewhere [1]. In short, milk samples from cases of CM were collected by field veterinarians in 51 veterinary practices distributed all over Sweden during a one-year period. The samples were cultured at 37°C for 16–24 hours on blood agar plates and evaluated in accordance with the routines of the veterinary practices. Cultured plates were sent to the National Veterinary Institute, Uppsala, Sweden where the growth was verified by routine laboratory tests. The time from day of sampling until arrival at the laboratory was 3.0 days (range 1–7 days), on average. Streptococci were identified by colony morphology, CAMP-reaction, and 12 biochemical reactions (hippurate, aesculine, salicine, sorbitol, mannitol, raffinose, lactose, saccharose, inuline, trehalose, starch and glycerine). For suspected *S. dysgalactiae* isolates not identified by the 12 biochemical reactions, Lancefield grouping (Streptex, Murex Biotech Limited, Dartford, UK) was used. To differentiate enterococci from *S. uberis*, growth of red colonies on SlaBa plates (Slanetz & Bartley Medium, Oxoid Ltd., Basingstoke, England) was evaluated. Isolates were stored frozen in trypticase soy broth containing 15% glycerol.

### Preparation and digestion of bacterial DNA

Isolates were thawed and cultured overnight at 37°C on 5% bovine blood agar supplemented with 0.05% esculine. One micro liter of *S. dysgalactiae* colony material was suspended in 250 μl lysis buffer (1 M NaCl, 10 mM Tris [pH 8.0], 200 mM EDTA, 0.5% Sarcosyl, 0.2% natrium deoxycholate; National Veterinary Institute, Uppsala, Sweden), mixed with 250 μl molten Agarose Prep (Amersham Biosciences, Uppsala, Sweden) and poured into plug molds (BioRad, CA, USA). Once solidified the plugs were placed in a buffer containing 5 ml lysis buffer and 200 μl lysozyme (from a stock solution of 20 mg/ml; Roche Diagnostics Scandinavia AB, Bromma, Sweden) and gently shaken overnight at 37°C. The buffer was then replaced by a proteolysis buffer containing 2.5 ml lysis buffer and 100 μl Proteinas K (from a stock solution of 50 U/ml; Roche Diagnostics Scandinavia AB, Bromma, Sweden) and the plugs were incubated for approximately 24 h at 56°C with gentle shaking. Plugs were washed twice with 10 ml Super-Q-water (National Veterinary Institute, Uppsala, Sweden) for 20 min in 56°C, and four times with 10 ml TrisEDTA (TE) buffer (10 mM Tris [pH 8.0], 1 mM EDTA; National Veterinary Institute, Uppsala, Sweden) for at least 20 min in 56°C. For long term storage, plugs were kept at 4°C in 1 ml TE buffer. Before digestion, plugs were sliced in halves, and one half was equilibrated in 200 μl CutSmart buffer (NewEngland Biolabs, MA, USA) for 30 min on gentle shaking at 25°C. The rest of the plug was returned to long term storage in TE buffer. After equilibration, each plug was digested at 25°C overnight in a digestion solution of 100 μl CutSmart buffer and 10 U of the restriction enzyme *SmaI* (NewEngland Biolabs, MA, USA).

Preparation and digestion of *S. uberis* DNA was performed using the same protocol as for *S. dysgalactiae* DNA, with the exception that digestion was performed in a digestion solution containing 5 U of *SmaI*.

### Pulsed-field gel electrophoresis

DNA fragments were separated using a clamped homogenous electric field device (CHEF-DR II, BioRad) with pulse times of 5–15 sec over 10 h, and 15–60 sec over 13 h at 6 V, and run through a 1.2% gel of Agarose NA (GE Healthcare, Uppsala, Sweden) in 0.5 × Tris-borat EDTA-buffer (0.9 M Tris-borat, 20 mM EDTA; National Veterinary Institute, Uppsala, Sweden). Then, gels were stained with GelRed (Biotium, CA, USA).

For each bacterial species, one isolate with an easily interpreted pattern was selected among our collected isolates, as an internal reference and was run every 5^th^ to 6^th^ lane on all gels. In addition, a Lambda Ladder PFG Marker (New England Biolabs, MA, USA) was used in the first and last lane on each gel.

The protocol was repeated for untypeable isolates.

### Dendrogram analysis

Macrorestriction patterns of *S. dysgalactiae* and *S. uberis* were analyzed separately. The dendrogram analyses were performed using BioNumerics software (BioNumerics Version 7.1; Applied Maths, Inc 2014; Austin, TX, USA). Similarity was computed using the Dice coefficient and an unweighted pair group method with arithmetic mean (UPGMA), with optimization set to 1.5% and the tolerance value set to 1.25%. Isolates were considered to be of the same cluster if the similarity level was above 80%, and of the same pulsotype when banding patterns were identical. *S. dysgalactiae* clusters were identified by capital letters, and pulsotypes within cluster with a number suffix (e.g. E1, E2, F1, F2). *S. uberis* clusters were identified by Roman numerals. Only clusters and pulsotypes represented by more than one isolate per genotype received an identity. The remaining genotypes were referred to as “singles”.

### Data editing and statistical analyses

#### Prevalence of genotypes

In order to study the presence and distribution of different genotypes on a national level and to ensure epidemiological independence among isolates, the first isolate per species from a monoinfected cow (i.e. cows where only one bacterial species was found, and where only one udder quarter was infected) collected from each herd was included. However, if a farm had no samples collected from a monoinfected cow, the first isolate per species collected from that herd was included. Given those criteria, 135 isolates of *S. dysgalactiae* and 103 isolates of *S. uberis* were included in this part of the study. Descriptive statistics were used to present prevalence of genotypes.

#### Differences in cow factors, season and geography

Differences in breed of cow, parity, days in milk (DIM), season and geographic region of Sweden between *S. dysgalactiae* and *S. uberis* cases were presented using descriptive statistics.

Breed of cow was divided into three categories: Swedish Holstein (SH), Swedish Red and White (SR) and mixed breed/other breed (where the most common observation was a mix between SH and SR).

Parity was categorized into first, second, third, and fourth and higher lactations. Stage of lactation was categorized into first month after calving or later. Seasons were categorized into pasture season (May to August), early housing season (September to December) and late housing season (January to April). The highest subdivision of geographic regions of Sweden (eastern Sweden, southern Sweden including the islands, and northern Sweden) according to the Nomenclature of Territorial Units for Statistics (NUTS1) was used when studying geographic occurrence of strains and genotypes [25]. Associations between bacterial species and categorical variables were investigated using Fisher’s exact test, as were associations between bacterial genotypes and categorical variables.

#### Outcome measurements and cow records

To investigate the impact of bacterial species or genotype on outcome of VTCM, only isolates from monoinfected cows were included (*S. dysgalactiae*: n = 98; *S. uberis*: n = 73). Cow composite SCC and milk yield at test milking, recurring or new cases of VTCM (VTCMadd), and culling due to mastitis during the follow-up period were used as outcome measurements. The follow-up period was zero to 120 days after VTCM for the parameters SCC, milk yield, and culling due to mastitis, and fourteen to 120 days for VTCMadd. Day zero was defined as the day the cultured plate arrived at the National Veterinary Institute. Somatic cell count and milk yield were measured at monthly milk recordings, and this data was obtained from the Swedish Official Milk Recording Scheme (SOMRS; Swedish Dairy Association, Stockholm, Sweden).

From SOMRS, cow records including breed, parity, date of calvings, and date and cause for culling were also obtained. The criterion for culling due to mastitis was fulfilled if either the primary or secondary officially recorded reason for culling was mastitis or increased SCC. Disease recordings were collected from the Swedish Animal Disease Recording System (SADRS) through SOMRS. A record in the SADRS of the original VTCM was present for 92% of the cows. VTCMadd was defined as an additional record of VTCM in the SADRS during the follow-up period.

Breed, parity and calving date was also recorded in a questionnaire by the veterinarian at milk sample submission. All cows where SOMRS records and questionnaire records were consistent were eligible for inclusion in the study. In case of minor discrepancies between official cow records and questionnaire records, individual assessment of the cow’s records was made before a decision was made to include or exclude the cow. In general, the official records were considered more likely to be correct. In cases of major discrepancies, the cow was included using questionnaire records only in the prevalence and risk factor part of the study, and was excluded completely from the outcome part of the study.

Eight cows were dried off or calved during the follow-up period. For these cows, only data from monthly milk recordings before dry-off was included.

Differences in outcome were calculated between the two different streptococci, between different pulsotypes and clusters within species and between groups of pulsotypes and clusters within species based on prevalence. Definitions of groups used has previously been described in Lundberg *et al*. [26].

Differences in SCC and milk yield between bacterial species, between genotypes or between groups of genotypes were first tested in univariable mixed-effect linear regression models with repeated measurements of monthly SCC or milk yield within cow during the follow-up period as outcome variable. Mixed-effect models were used to take into account that repeated measurements of SCC or milk yield for a certain cow might be more similar than measurements of SCC or milk yield for different cows, hence, the random effect in the mixed models was repeated measurement of SCC or milk yield and an independent covariance structure (equal variances for random effects, all covariances are zero) was used. To obtain normally distributed residuals, SCC was transformed using the Box-Cox power transformation (SCC ^-0.1263839^-1)/-0.1263839. Bacterial species or genotype (pulsotypes, clusters, or groups of pulsotypes or clusters) were used as explanatory variables to the outcome. The cow parameters breed, parity and DIM at monthly milk recordings were included in the models as independent variables because of their possible impact on SCC and milk yield. When differences in milk yield were tested, SCC was included among independent variables and vice versa. To reduce the full models, a manual stepwise backward model selection procedure was used and only variables with a p-value of ≤0.05 were included in the final models. Two-way interactions between the significant main effects were tested. Possible confounders were considered in all models. A variable was considered as a confounder if the point estimates of the coefficients in a model changed >20% with the potential confounder present. The model fit of the multivariable analyses was tested by visual examination of diagnostic plots according to Dohoo *et al*. [27].

In addition, the proportions of cows with a SCC below 200 000 cells/ml at all monthly milk recordings during the follow-up period was calculated for *S. dysgalactiae* and *S. uberis* as well as for different genotypes or genotype groups within species. The follow-up period was shortened to 14 to 120 days to allow for SCC to normalize after therapy, and differences in proportions were tested with the Fisher’s exact test. Only cows with data recorded for at least two monthly milk recordings were included in these calculations.

Proportions of cows with a VTCMadd registered and proportion of cows that were culled due to mastitis during the follow-up period were presented using descriptive statistics.

All statistical analyses were performed using Stata 13 (StataCorp, 2014; Stata Statistical Software: Release 13.1; College Station, TX, USA: StataCorp LP).

## Results

### Macrorestriction patterns and prevalence of genotypes

One hundred thirty two of the *S. dysgalactiae* isolates and 97 of the *S. uberis* isolates could be genotyped using our protocol.

**Figure 1:**
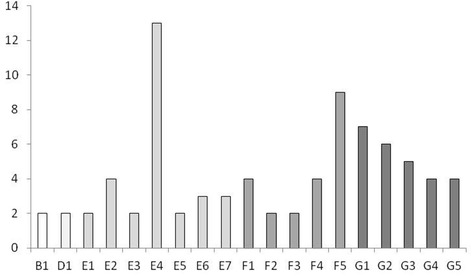
**Numbers of** ***Streptococcus dysgalactiae*** **isolates of different pulsotypes.** Numbers of epidemiologically independent *Streptococcus dysgalactiae* isolates of different pulsotypes from cases of veterinary-treated clinical mastitis in Sweden (pulsotypes only isolated once (n = 52) are not included). Pulsotypes belonging to the same cluster share the same color.

Pulsed-field gel electrophoresis of *S. dysgalactiae* yielded 7 to 14 fragments in the considered size range of 45 to 500 kb. (Additional file 1) shows a dendrogram of the results. Analysis of the *S. dysgalactiae* isolates revealed 71 pulsotypes. Nineteen of the pulsotypes could be found in two to 13 herds each (Figure 1). The remaining 52 pulsotypes were only found once each in the material. Sixty-eight of the pulsotypes could be compiled into nine clusters. Three pulsotypes could not be clustered with other pulsotypes. Six of the clusters were considered rare, each represented by two to six isolates, while three of the clusters (E, F, and G) were considered common, each represented by 30 to 40 isolates. Those three clusters accounted for 82% of the isolates (Table 1).

**Table 1:** **Numbers (n) and percentages (%) of epidemiologically independent** ***Streptococcus dysgalactiae*** **isolates from cases of veterinary-treated clinical mastitis in Sweden divided into clusters after genotyping by pulsed-field gel electrophoresis and clustering at the level of 80% genetic similarity**

**Cluster**	**n (%)**
A	3 (2.3)
B	6 (4.6)
C	2 (1.5)
D	5 (3.8)
E	40 (30.3)
F	30 (22.7)
G	38 (28.8)
H	3 (2.3)
I	2 (1.5)
Singles	3 (2.3)
Total	132 (100)

Pulsed field gel electrophoresis of *S. uberis* yielded 10 to 13 fragments in the 40–660 kb size range. (Additional file 2) shows a dendrogram of the results. All 97 *S. uberis* isolates were found to be of different pulsotypes. Forty-five isolates belonged to 21 clusters; the remaining isolates could not be assigned to a cluster (Additional file 2). Each cluster was represented by two or three isolates.

### Differences in cow factors, season and geography between *S. dysgalactiae* and *S. uberis*

Descriptive statistics are given in Table 2. A larger proportion of *S. uberis*-isolates than of *S. dysgalactiae*-isolates came from cows of the SH breed compared to cows of the SR breed, but the difference was not statistically significant (*P* = 0.079). The distribution of parities and stage of lactation was similar among *S. dysgalactiae* and *S. uberis* isolates. About half of the isolates came from cases of VTCM occurring during the first month of lactation, with the rest spread out throughout the lactation.

**Table 2:** **Numbers (n) and percentages (%) of** ***Streptococcus dysgalactiae*** **and** ***Streptococcus uberis*** **isolates from cases of veterinary-treated clinical mastitis in cows of different breeds, parities, and stages of lactation, occurring in different seasons and geographic regions of Sweden**

**Variable**	**Class**	***S. dysgalactiae*** **, n (%)**	***S. uberis*** **, n (%)**
Breed^1^	SR	67 (49.6)	38 (36.9)
	SH	62 (45.9)	58 (56.3)
	Other	6 (4.4)	7 (6.8)
Parity	First	56 (41.5)	42 (40.8)
	Second	30 (22.2)	22 (21.4)
	Third	21 (15.6)	18 (17.5)
	Fourth or higher	28 (20.7)	21 (20.4)
Stage of lactation	<30 days postpartum	68 (50.4)	44 (42.3)
	≥30 days postpartum	67 (49.6)	59 (57.3)
Season	Early housing^2^	48 (35.6)	43 (41.8)
	Late housing^3^	47 (34.8)	23 (22.3)
	Pasture^4^	40 (29.6)	37 (35.9)
Region	Southern Sweden	91 (67.4)	59 (57.3)
	Eastern Sweden	26 (19.3)	21 (20.4)
	Northern Sweden	18 (13.3)	23 (22.3)

^1^SR = Swedish Red, SH = Swedish Holstein; ^2^September–December; ^3^January–April; ^4^May–August.

There was a significant difference in occurrence of *S. dysgalactiae* and *S. uberis* over seasons (*P* <0.05; Table 2). The occurrence of *S. uberis* was lowest during the late housing season; no fluctuations throughout the year could be seen for *S. dysgalactiae*.

**Figure 2:**
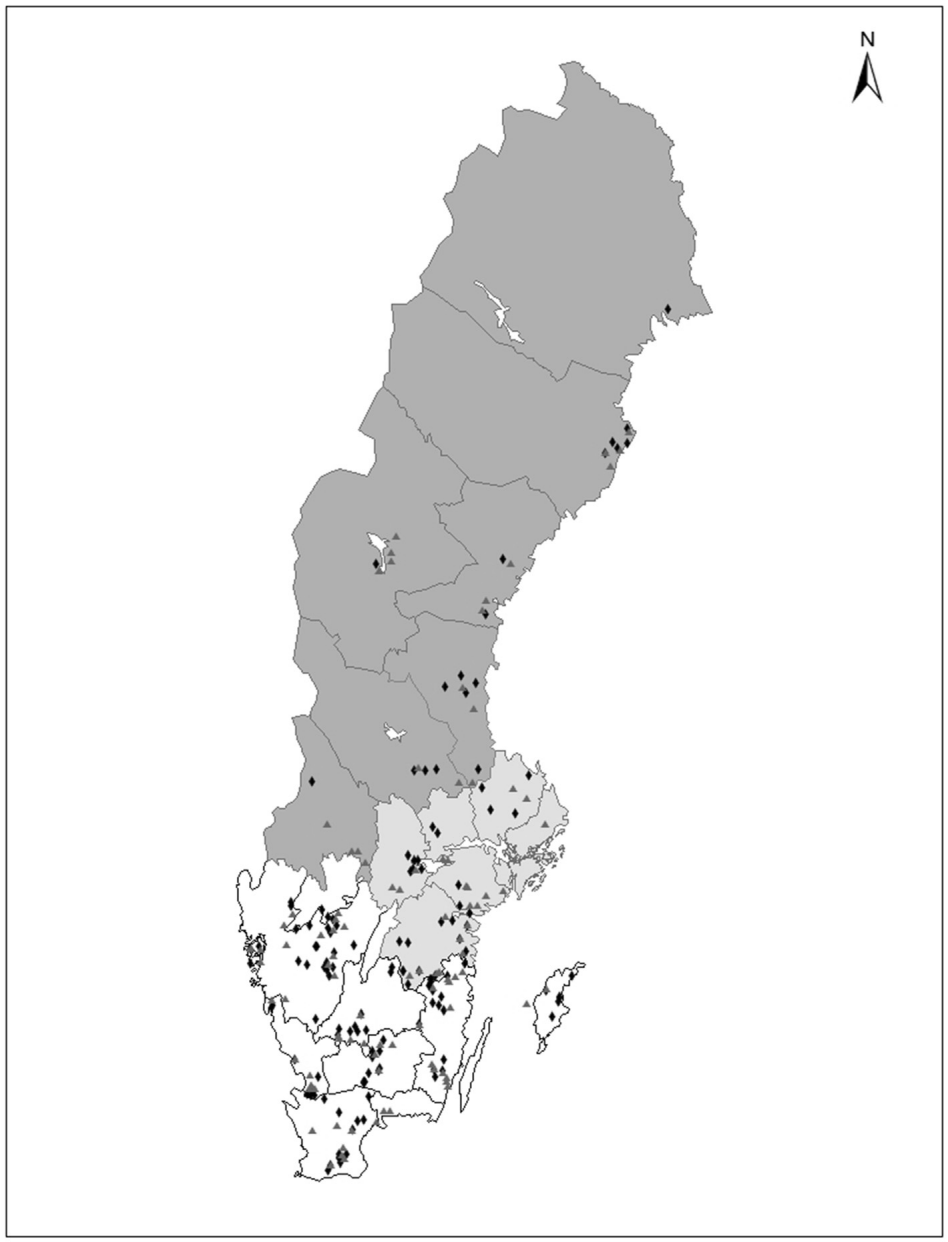
**Geographic distribution of cases of clinical mastitis.** Geographic distribution of cases of veterinary-treated clinical mastitis caused by *Streptococcus dysgalactiae* (black diamonds) and *Streptococcus uberis* (grey triangles).

The majority of both *S. dysgalactiae* and *S. uberis* isolates came from Southern Sweden. A larger proportion of *S. uberis*-isolates than of *S. dysgalactiae*-isolates came from Northern Sweden, and the opposite was found for Southern Sweden, but the difference was not significant (*P* = 0.08). The geographic distribution of the isolates within the country is given in Figure 2.

### Differences in cow factors, season and geography between bacterial genotypes within species

For *S. dysgalactiae*, the number of isolates per pulsotype and most clusters were small, and statistical analyses of differences between groups were therefore not performed. Some pulsotypes could, however, only be found in one breed, lactation stage or parity, or during one season or in a specific region. The distribution of clusters, or groups of pulsotypes or clusters, did not indicate any trends related to cow factors, season or geography.

Due to few cows in each *S. uberis* cluster, statistical analyses of differences between clusters were not performed and no trends could be identified.

### Differences in outcome between *S. dysgalactiae* and *S. uberis*

For 163 monoinfected cows (95 *S. dysgalactiae*-cows and 68 *S. uberis*-cows) results from at least one monthly milk recording after the VTCM were available. In total, 541 monthly SCC and milk yield recordings were included (3.3 recordings per cow, on average).

**Figure 3:**
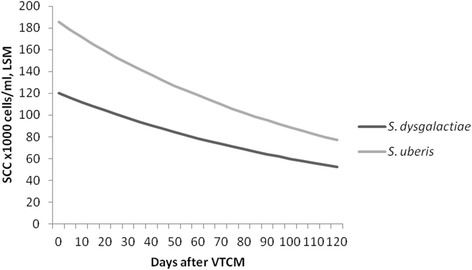
**Long-term outcome after clinical mastitis as measured by SCC.** Least square means (LSM) of cow somatic cell counts (SCC; ×1 000 cells/ml) at monthly milk recordings after veterinary-treated cases of clinical mastitis (VTCM) from the results of a multivariable mixed-effect linear regression model investigating associations between *Streptococcus dysgalactiae* and *Streptococcus uberis*.

During the follow-up period after the VTCM, the SCC of *S. dysgalactiae*-cows was significantly lower than the SCC of *S. uberis*-cows (p <0.05; Figure 3). The median SCC during the follow-up period was 71 500 cells/ml (1^st^ quartile: 35 500; 3^rd^ quartile: 203 500) and 108 000 cells/ml (1^st^ quartile: 47 000; 3^rd^ quartile: 319 000 for *S. dysgalactiae*-cows and *S. uberis*-cows, respectively. The difference in SCC between species remained in the multivariable model when the influence of milk yield, parity, breed and DIM on SCC also was considered (Table 3).

**Table 3:** **Final hierarchical multivariable linear-regression analysis of variables significantly (** ***P*** **<0.05) associated with somatic cell count (SCC; transformed using the BoxCox power transformation) at monthly milk recordings during a follow-up period of 120 days after a veterinary treated clinical mastitis**

**Variable** ^**1**^	***β***	**S.E. (** ***β*** **)**	***P*** **-value**	**LSM** ^**2**^	**95% CI (LSM)**
Intercept	3.996	0.17	-	-	-
**Bacterial finding**					
*S. dysgalactiae*	Ref.^3^	-	-	86.6	70.1, 107.5
*S. uberis*	0.260	0.093	0.005	138.6	106.3, 182.6
**Other factors**					
Parity					
First	Ref.	-	-	75.9	59.8, 97.1
Second	0.219	0.113	0.051	112.0	82.8, 153.4
Third	0.169	0.137	0.218	102.3	68.7, 155.6
Fourth or higher	0.433	0.136	0.001	260.5	109.1, 260.5
Milk yield	−0.002	0.0005	0.000	-	-
At Q1: 23.8 kg	-	-	-	132.8	108.1,164.2
At Q2: 28.7 kg	-	-	-	109.8	92.0, 131.4
At Q3: 33.4 kg	-	-	-	91.8	76.3, 111.0
Days in milk	−0.001	0.0004	0.001	-	-
At Q1: 67 days	-	-	-	129.4	105.0, 160.5
At Q2: 115 days	-	-	-	115.6	96.4, 139.3
At Q3: 188 days	-	-	-	97.8	81.5, 117.7

^1^Q1, Q2, and Q3 = first quartile, second quartile/median, and third quartile, respectively.

^2^Least square means (LSM) of Box-Cox transformed SCC back-transformed to original scale (×1 000/ml) presented by variable.

^3^Ref. = Reference category.

Eighty-four *S. dysgalactiae*-cows and 58 *S. uberis*-cows had more than one registered recording of SCC during the period of 14 to 120 days after VTCM. The proportion of *S. dysgalactiae*-cows with a SCC below 200 000 cells/ml at all recordings during this period was 58%. The corresponding percentage for *S. uberis*-cows was 43%. The difference between species was not significant (*P* = 0.09).

The average daily milk yield at monthly milk recordings during the follow-up period was 28.5 kg (range: 9.2–50.8 kg) and 29.3 kg (range: 9.8–53.6 kg) for *S. dysgalactiae*-cows and *S. uberis*-cows, respectively, and did not differ significantly between species.

Eleven (12%) *S. dysgalactiae*-cows had a VTCMadd registered and six (6%) were culled during the follow-up period. The corresponding numbers for *S. uberis*-cows were six (9%) with VTCMadd and six (8%) culled.

### Differences in outcome between bacterial genotypes within species

No univariable associations between milk yield or SCC and bacterial species or genotype or groups of genotypes could be found. However, there seemed to be some indication that the proportion of *S. dysgalactiae* cows having SCC constantly below 200 000 cells/ml during the follow-up period varied between pulsotypes (Table 4), although the numbers of isolates in each group were too small for statistical analyses.

**Table 4:** **Numbers of cows with a somatic cell count (SCC) below 200 000 cells/ml at all monthly milk recordings, and number of cows with SCC above 200 000 cells/ml at least one monthly milk recording 14 to 120 days after veterinary-treated clinical mastitis, caused by different clusters or pulsotypes1 of** ***Streptococcus dysgalactiae***

**Cluster/Pulsotype**	**<200 000 cells/ml**	**>200 000 cells/ml**	**Total**
A	2	1	3
B	2	3	5
B1	0	2	2
D	1	1	2
E	14	10	24
E2	1	2	3
E4	5	2	7
E5	1	1	2
E6	1	2	3
E7	2	1	3
F	11	6	17
F1	1	2	3
F4	4	0	4
F5	5	1	6
G	13	12	25
G1	1	5	6
G2	3	1	4
G3	2	1	3
G4	1	2	3
G5	2	1	3
H	1	1	2

^1^Cows infected with unique clusters/pulsotypes are not included in the table.

The number of cows within each pulsotype or cluster of *S. dysgalactiae* and *S. uberis*, or within each group of pulsotypes or clusters, that had a VTCMadd registered or that were culled during the follow-up period was too small for statistical analysis and no trends could be identified.

## Discussion

### Genotype distribution

In line with most previous studies [2,3,5-7], we found that the genotype pattern of *S. uberis* is heterogeneous in Sweden and we found no evidence of contagious spread of *S. uberis* between herds as all isolates investigated were of different pulsotypes. In contrast, identical pulsotypes of *S. dysgalactiae* were found in multiple herds in the present study, although 39% of the isolates belonged to unique pulsotypes. The findings of identical strains of *S. dysgalactiae* in different herds is in line with previous studies [5,6,11]. A common environmental source could explain why the same genotype was found in multiple herds, but this hypothesis seems less likely for many of the genotypes since identical isolates were found in separate parts of the country. Trade of infected animals is another, and perhaps more likely, way of spread of the pathogen between herds and it has been shown that the trade of livestock is expansive within Sweden [28]. For local spread (kilometers) the fly *Hydrotaea irritans*, which can act as a vector for *S. dysgalactiae* [29], could also be involved. It is possible that some genotypes contain virulence factors that make them especially apt to spreading between cows. No such attributes of the strains were investigated in this study, but previous studies have shown strain differences in virulence factors, possibly connected to spread between cows, for both *S. dysgalactiae* and *S. uberis* [30].

Given the existing information and the original study design, it was not possible to study the genetic within-herd variation among the udder pathogens. Previous studies of both *S. dysgalactiae* and *S. uberis* have, however, shown that several strains can be found in a herd and that in some instances one or a few of these strains can be found in other herds as well [4,6,10]. It is therefore possible that we, when including only one sample per herd, might have underestimated the spread of genotypes between herds for both *S. dysgalactiae* and *S. uberis* in the present study.

The moderate and large genotypic variation found for *S. dysgalactiae* and *S. uberis*, respectively, in the present study differs markedly from a previous study on isolates of *Staphylococcus aureus* from the same national survey [26]. In that study, almost two thirds of the *S. aureus* isolates belonged to two pulsotypes, emphasizing the contagious nature of *S. aureus* as opposed to the two streptococcal species investigated in the present study.

### Differences in outcome of VTCM between bacterial species and genotypes

Cows veterinary-treated for CM caused by *S. dysgalactiae* had a lower SCC during the follow-up period, than did cows treated for infections caused by *S. uberis*. Possible explanations for this finding could be a stronger inflammatory response to *S. uberis* at the initial infection [31] or difference in bacteriological cure rates between species [19].

In the present study, we did not have any information on SCC at the time of VTCM other than the California Mastitis Test (CMT) score performed by the investigating veterinarian. The CMT is, however, an imprecise test, and most cases of CM are categorized as either 4 or 5 on the Nordic scale (i.e. SCC > ~800 000 cells/ml) making CMT unsuitable for use in the present study.

Unfortunately, no information about bacteriological cure after VTCM was available in our study material. National guidelines at the time of sampling stated that infections during lactation caused by *S. dysgalactiae* and *S. uberis* should be treated by parenteral benzyl penicillin for 4–5 days [32]. The isolates in the present study were tested for susceptibility to penicillin *in vitro* [33]. All of the *S. dysgalactiae* isolates and 94% of the *S. uberis* isolates tested were susceptible, therefore it seems unlikely that any possible difference in bacteriological cure was due to resistance to penicillin.

No statistically significant differences in SCC between genotypes, or groups of genotypes, within bacterial species could be found in this study. The number of genotypes in relation to the total number of isolates was high, which made comparisons difficult. The discriminatory power of PFGE is high, making it probable that isolates with identical PFGE patterns are in fact genetically related. Tenover *et al*. [34] proposed that genotypes with a one to three band difference (consistent with one genetic event) are closely related and that genotypes with a four to six band difference are possibly related. This was, however, proposed for hospital outbreaks and to our knowledge, similar guidelines for national surveillance material do not exist. It is therefore not certain that isolates with the 80% level of genetic similarity used for clustering in the present study are related. This could explain why differences in our outcome parameters could not be found between *S. dysgalactiae* clusters. To further study if differences in outcome of VTCM could be attributed to bacterial genotypes of *S. dysgalactiae* or *S. uberis*, a study including a larger number of natural infections could be used, resulting in more isolates per pulsotype or cluster and enabling statistical comparisons. In experimental infections, significant differences in pathogenicity between different strains of *S. dysgalactiae* [35], as well as between different strains of *S. uberis* [36], have been shown in the past, and another option may be to study long-term outcome after experimental infections of isolates of different genotypes. However, it is also possible that isolates of the same pulsotype or cluster express different virulence factors. Further studies in this area are therefore warranted.

We found no differences in milk yield between bacterial species, or between genotypes within species. As milk yield and SCC are correlated, i.e. the milk yield decreases with increasing SCC [37] and we found a difference in SCC between streptococcal species, a difference in milk yield between *S. dysgalactiae* and *S. uberis* during the follow-up period would have been expected. It is possible; however, that the difference in SCC between species was too small to influence the milk yield significantly. It is also possible that *S. uberis* infections are more common in high yielding cows, but since information on milk yield before VTCM was not available in the present study this could not be controlled for in the comparison between bacterial species.

### Species and genotype differences in cow factors, season and geography

The results indicate that breed, season and geographical region, but not parity and stage of lactation, might differ between cases of VTCM due to *S. dysgalactiae* and *S. uberis* in Sweden. However, the results should be interpreted with caution as the study was not designed specifically for evaluation of the effect of cow factors, season and geography on the risk for infection with different streptococci. For example, information about other, possibly confounding, factors, like herd size, barn type and breed distribution in herds and over geographic regions, were not available in this study and thus could not be controlled for in the statistical analysis. The study material was, however, representative for VTCM on a national level as the selection of cases was based on the distribution of cows throughout the country.

The lower and higher proportion of *S. uberis* in the late housing season and in SH cows, respectively, compared to *S. dysgalactiae* is in line with the previous study where the occurrence of VTCM caused by *S. uberis* was compared to VTCM caused by all other pathogens [1]. The seasonal pattern is also similar to the results of a Canadian study, where the incidence rate of CM caused by *S. uberis* was low during the winter and peaked in August [38]. However, in Canada there seemed to be a decline in *S. dysgalactiae* incidence rate during the summer as well [38], a pattern not seen in our material. Moreover, regional differences have been described for *S. dysgalactiae* in Canada [24], but have to our knowledge not been described for *S. uberis*.

As already mentioned, parity did not differ between cases of *S. dysgalactiae* and *S. uberis* mastitis. Unpublished observations suggest, however, that mastitis cases due to the two streptococcal species investigated in the present study are more prevalent in first parity cows compared to clinical cases of *Staphylococcus aureus* mastitis studied in a previous paper [26]. The CM cases of the two studies originated from the same national survey.

The effect of DIM on the distribution of CM caused by *S. uberis* in this study seem to differ from that reported from New Zealand where *S. uberis* is predominantly isolated during the first month of lactation [39]. These differences between countries may be due to different production systems.

## Conclusions

Identical *S. dysgalactiae* genotypes were found in cases of bovine CM from more than one herd, indicating some spread of infections between Swedish dairy herds, but a large proportion of the banding patterns were only found once. In contrast, we found no evidence of spread of *S. uberis* genotypes between herds. The genetic variation among *S. uberis* isolates was substantial. The results suggest that, in Sweden, *S. uberis* should be considered as an environmental pathogen, while *S. dysgalactiae* probably can act either as a contagious or an environmental pathogen, at least when considering spread between herds. The only outcome of veterinary-treated CM that differed significantly between bacterial species or genotypes was the milk SCC, which was lower during a four month follow-up period after veterinary treatment of CM if *S. dysgalactiae* rather than *S. uberis* was isolated from the case.

## Additional files

Additional file 1:
**Dendrogram of** ***S. dysgalactiae*** **.** Dendrogram of pulsed-field gel electrophoresis profiles of epidemiologically independent *Streptococcus dysgalactiae* isolates collected from cases of veterinary-treated clinical mastitis in Sweden. Isolate code (IDnr.), cluster name (Cl.) and pulsotype names (PT) are shown, as well as the 80% cutoff line (vertical line) for clusters. Additional file 2:
**Dendrogram of** ***S. uberis*** **.** Dendrogram of pulsed-field gel electrophoresis profiles of epidemiologically independent *Streptococcus uberis* isolates collected from cases of bovine clinical mastitis in Sweden. Isolate code (IDnr.) and cluster (Cl.) names are shown, as well as the 80% cutoff line (vertical line) for clusters.

## Abbreviations

NUTS: Nomenclature of Territorial Units for Statistics
PFGE: Pulsed-field gel electrophoresis
SCC: Somatic cell count
SOMRS: Swedish Official Milk Recording Scheme
SADRS: Swedish Animal Disease Recording System
VTCM: Veterinary-treated clinical mastitis
VTCMadd: Additional episodes of VTCM for the same cow
