# Supplementary figures and images for: Prevalence of bacterial genotypes and outcome of bovine clinical mastitis due to Streptococcus dysgalactiae and Streptococcus uberis
Source: Acta Vet Scand. 2014 Nov 27;56(1):80. doi: 10.1186/s13028-014-0080-0 (PMC4255449; doi:10.1186/s13028-014-0080-0)

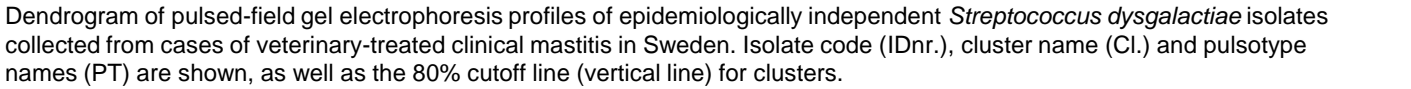

Supplement: Additional file 1: — Dendrogram of S. dysgalactiae . Dendrogram of pulsed-field gel electrophoresis profiles of epidemiologically independent Streptococcus dysgalactiae isolates collected from cases of veterinary-treated clinical mastitis in Sweden. Isolate code (IDnr.), cluster name (Cl.) and pulsotype names (PT) are shown, as well as the 80% cutoff line (vertical line) for clusters. [file 13028_2014_80_MOESM1_ESM.pdf]
