# Supplementary material for: Prevalence of bacterial genotypes and outcome of bovine clinical mastitis due to Streptococcus dysgalactiae and Streptococcus uberis
Source: Acta Vet Scand. 2014 Nov 27;56(1):80. doi: 10.1186/s13028-014-0080-0 (PMC4255449; doi:10.1186/s13028-014-0080-0)
Supplement: Additional file 2: — Dendrogram of S. uberis . Dendrogram of pulsed-field gel electrophoresis profiles of epidemiologically independent Streptococcus uberis isolates collected from cases of bovine clinical mastitis in Sweden. Isolate code (IDnr.) and cluster (Cl.) names are shown, as well as the 80% cutoff line (vertical line) for clusters. [file 13028_2014_80_MOESM2_ESM.pdf]

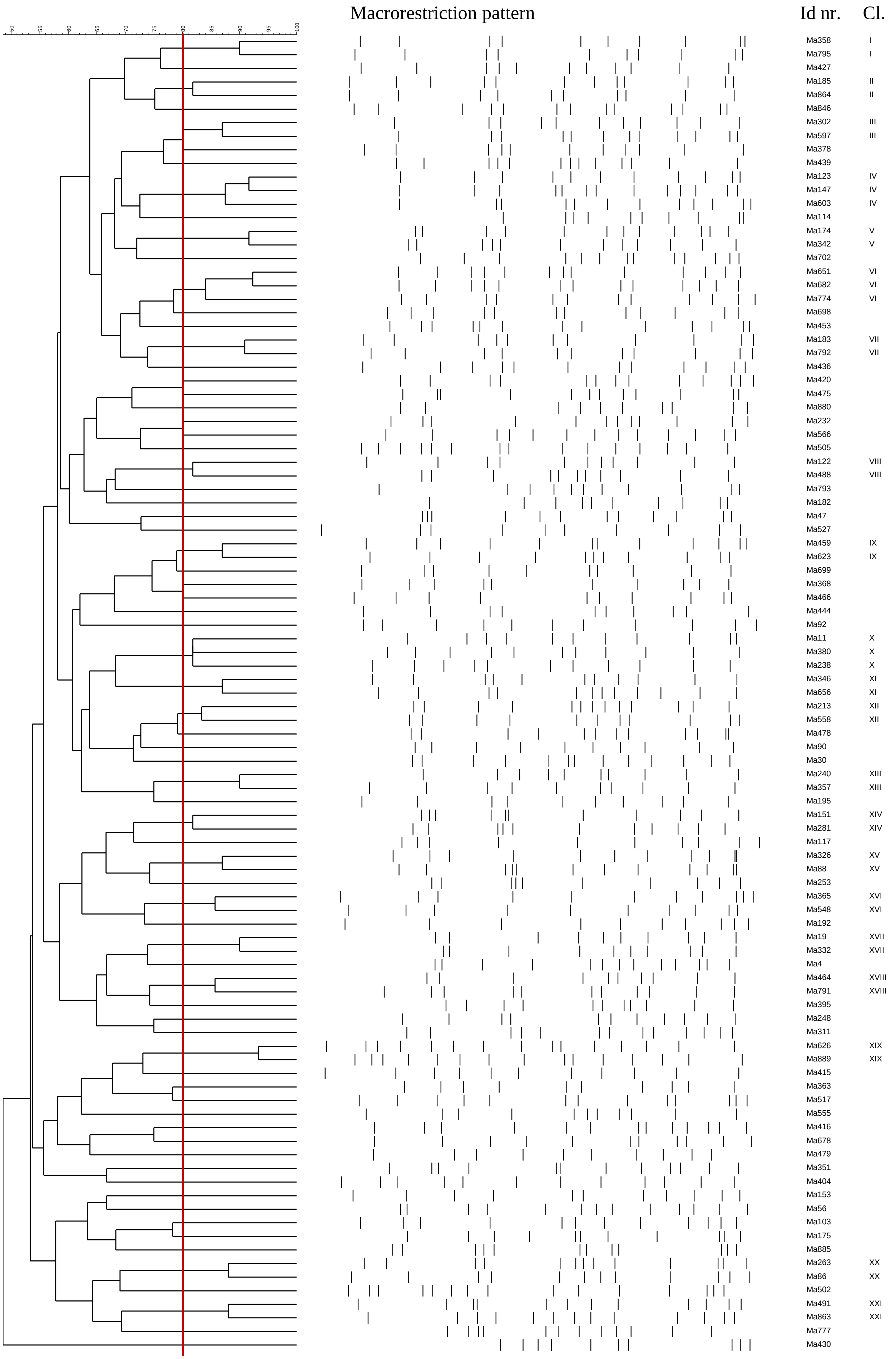

Dendrogram of pulsed-field gel electrophoresis profiles of epidemiologically independent *Streptococcus uberis* isolates collected from cases of bovine clinical mastitis in Sweden. Isolate code (IDnr.) and cluster (Cl.) names are shown, as well as the 80% cutoff line (vertical line) for clusters.
